# Interactions between a pathogenic *Blastocystis* subtype and gut microbiota: in vitro and in vivo studies

**DOI:** 10.1186/s40168-019-0644-3

**Published:** 2019-03-11

**Authors:** John Anthony Yason, Yi Ran Liang, Chin Wen Png, Yongliang Zhang, Kevin Shyong Wei Tan

**Affiliations:** 1 Laboratory of Molecular and Cellular Parasitology, Department of Microbiology and Immunology, Yong Loo Lin School of Medicine, National University of Singapore, 5 Science Drive 2, Singapore, 117545 Singapore; 2 Institute of Biology and Natural Sciences Research Institute, College of Science, University of the Philippines, Diliman, Quezon City, 1101 Philippines; 3 Microbiome Otago, Department of Microbiology and Immunology, University of Otago, PO Box 56 720, Cumberland St, Dunedin, 9054 Otago New Zealand

**Keywords:** *Blastocystis*, Subtypes, Gut microbiota, Dysbiosis, *Bifidobacterium*

## Abstract

**Background:**

*Blastocystis* is a common gut eukaryote detected in humans and animals. It has been associated with gastrointestinal disease in the past although recent metagenomic studies also suggest that it is a member of normal microbiota. This study investigates interactions between pathogenic human isolates belonging to *Blastocystis* subtype 7 (ST7) and bacterial representatives of the gut microbiota.

**Results:**

Generally, *Blastocystis* ST7 exerts a positive effect on the viability of representative gut bacteria except on *Bifidobacterium longum*. Gene expression analysis and flow cytometry indicate that the bacterium may be undergoing oxidative stress in the presence of *Blastocystis*. In vitro assays demonstrate that *Blastocystis*-induced host responses are able to decrease *Bifidobacterium* counts. Mice infected with *Blastocystis* also reveal a decrease in beneficial bacteria *Bifidobacterium* and *Lactobacillus*.

**Conclusions:**

This study shows that particular isolates of *Blastocystis* ST7 cause changes in microbiota populations and potentially lead to an imbalance of the gut microbiota. This study suggests that certain isolates of *Blastocystis* exert their pathogenic effects through disruption of the gut microbiota and provides a counterpoint to the increasing reports indicating the commensal nature of this ubiquitous parasite.

## Background

*Blastocystis* is a common gut eukaryote detected in human and many animal hosts [1, 2]. It is classified under the group Stramenopiles which mostly comprises unicellular flagellated or ciliated free-living organisms [2, 3]. *Blastocystis*, however, is an obligately anaerobic and parasitic protist and is transmitted via the fecal-oral route [1]. Estimates put the number of individuals infected by this parasite to more than 1 billion worldwide [4]. Although it is more common in developing countries, surveys in developed countries often indicate prevalence rates of more than 5% in the general population [5]. The role of *Blastocystis* in disease has been the subject of many investigations. There are studies associating it with symptoms of a gastrointestinal disease [6–8] while others could not find the basis for defining it as pathogenic [9, 10]. More recently, infection with *Blastocystis* has been linked with irritable bowel syndrome (IBS) [11] and inflammatory bowel disease (IBD) [12]. There are however conflicting reports on whether *Blastocystis* was really the sole causative agent in these cases [12–15]. In another perspective, IBS [16–18] and IBD [19, 20] were also linked to the disruption of the gut microbiota or dysbiosis. *Blastocystis’* role in IBS or IBD may thus be mediated by altering gut microbiota composition. However, the few microbiome studies on *Blastocystis* generally identified it as a common commensal in the human gut. These analyses associated the presence of *Blastocystis* with higher diversity of gut microbiota [21–24]. However, one study indicated that it caused a decrease in beneficial bacteria particularly *Bifidobacterium* and *Lactobacillus* spp. [25]. These discrepancies may be due to the complex nature of *Blastocystis* wherein several genetically distinct subtypes (ST) exist. Different *Blastocystis* STs could exhibit different growth rates, drug susceptibilities, host ranges, and other biological features [1, 26]. These differences could therefore influence the protist’s influence on the gut microbiota. Indeed, it has been suggested that microbiota composition in relation to *Blastocystis* may be dependent on the organism’s subtype identity [27].

With these in mind, the current study explored the interactions between a particular ST of *Blastocystis*, ST7, and prokaryotic representatives of the gut microbiota. ST7 isolates possess pathogenic properties not observed in other STs. For example, drug susceptibility assays indicated that ST7 isolates are resistant to metronidazole, the usual drug of choice to clear protistan parasites [28]. In vitro culture assays also revealed that ST7, but not ST4 isolates, could compromise the intestinal epithelial barrier [29]. In vivo experiments also revealed that isolates from this ST could cause tissue damage in the mouse intestines [30]. This ST appears rarely in surveys but has been reported to be strongly associated with gastrointestinal symptoms [31]. We used co-culture experiments to determine the effect of *Blastocystis* ST7 isolates on the viability of select gut bacteria representatives. Biological assays as well as gene expression analyses were used to investigate a possible mechanism on how *Blastocystis* affect these bacterial populations. Lastly, we conducted in vivo experiments involving infection of mice with *Blastocystis* and subsequent analyses of bacterial content in the fecal samples. The results of this study indicated that *Blastocystis* can disrupt gut microbiota populations particularly decreasing the content of *Bifidobacteria* and *Lactobacillus* but increasing *Escherichia coli*. Possible explanations of these occurrences point to oxidative stress caused by *Blastocystis* as well as host factors induced by the parasite. Our data indicates that while *Blastocystis* spp. may be a member of healthy gut microbiota, specific isolates or rare ST may disrupt homeostasis leading to pathological states in the host.

## Methods

### Blastocystis cultures

Human *Blastocystis* isolates were acquired from patients at the Singapore General Hospital in the early 1990s before the Institutional Review Board was established at the National University of Singapore (NUS). *Blastocystis* ST7 isolates B and H are maintained at a microbial collection at the Department of Microbiology and Immunology of the NUS. Both isolates ST7-B and ST7-H were axenized previously [32] and maintained in 8 ml pre-reduced Iscove’s modified Dulbecco’s medium (IMDM) (Gibco) supplemented with heat-inactivated 10% horse serum (Gibco). These were incubated in anaerobic jars (Oxoid) with Anaerogen gas packs (Oxoid) at 37 °C and subcultured every 3–4 days. *Blastocystis* cell counts were done manually using hemocytometer (Kova International).

### Bacterial cultures

*Escherichia coli* ATCC 11775, *Enterococcus faecalis* ATCC 29212, *Bacillus subtilis* ATCC 6633, and *Bacteroides fragilis* ATCC 25285 were cultured and maintained in Luria-Bertani (LB) broth and agar (Sigma). *Bifidobacterium longum* ATCC 15707 and *Lactobacillus brevis* ATCC 14869 were cultured and maintained in Bifidus selective medium (BSM) (Sigma) and deMan, Rogosa, Sharpe (MRS) medium (Sigma), respectively, in broth and agar forms. *B. fragilis* and *B. longum* were maintained in anaerobic condition inside anaerobic jars (Oxoid) with Anaerogen gas packs (Oxoid). All cultures were incubated at 37 °C. Absorbance readings of bacterial broth cultures prior to experiments were done using Tecan Infinite F200 microplate reader.

### Co-culture experiments

*Blastocystis* cells and bacterial cells were washed twice in phosphate-buffered saline (PBS) at 1000×*g* for 10 min. A concentration of 1 × 10^7^ cells/ml of *Blastocystis* ST7-B or ST7-H and 1 × 10^9^ CFU/ml of bacteria (*E. coli*, *E. faecalis*, *B. longum*, *L. brevis*, *B. subtilis*, or *B. fragilis*) was incubated for 24 h at 37 °C in pre-reduced PBS. Controls with only 1 × 10^7^ cells/ml *Blastocystis* and only 1 × 10^9^ CFU/ml bacteria, both re-suspended in pre-reduced PBS, were also incubated for 24 h at 37 °C. After 24 h, *Blastocystis* cells were counted using a hemocytometer (Kova International) after a 50-fold dilution of the neat cultures. A drop plate method was utilized for the enumeration of bacterial colony-forming units (CFUs) [33]. Bacterial colony-forming unit per milliliter was determined when the colonies appeared on the agar plates.

### *B. longum* ROS staining and flow cytometry

To determine cellular reactive oxygen species (ROS) content in *B. longum* cells, the stain 2′,7′–dichlorofluorescein diacetate (DCFDA) (Sigma) was used at a concentration of 20 μM for 30 min at 37 °C. Before the co-culture experiment, *B. longum* cells were stained with Baclight Red (Thermofisher) at a concentration of 1 μM for 15 min at room temperature to be able to gate for these cells in flow cytometry. The cells were run in Attune Nxt Flow Cytometer (Life Technologies) using blue (488 nm) and yellow (561 nm) lasers.

### *B. longum* oxidoreductases genes expression analysis

mRNA from *B. longum* cells were extracted using RNAzol RT (Sigma-Aldrich) following the manufacturer’s instructions. cDNA was synthesized using iScript cDNA kit (Bio-Rad). The gene expression of *Bifidobacterium* oxidoreductases were determined in a qPCR assay using primers reported previously [34]. SsoAdvanced™ Universal SYBR Green Supermix (Bio-Rad) was used and amplifications performed in an iCycler thermocycler with iQ5 attachment (Bio-Rad).

### HT-29 monolayer

Cells were maintained in T-75 flasks (Corning) in a humidified incubator with 5% CO_2_ at 37 °C. Culture medium consisted of 10% heat-inactivated FBS (Gibco) and 1% each of sodium pyruvate (Gibco), non-essential amino acids (Gibco), and penicillin-streptomycin in Dulbecco’s modified Eagle’s medium (DMEM) (Thermo Scientific). HT-29 cells were then used for co-culture experiments with *Blastocystis* and *B. longum* at 1 × 10^6^ cells/ml and 1 × 10^9^ CFU/ml, respectively.

### Epithelial permeability measurement

HT-29 Cells were seeded with complete medium onto Millicell hanging cell culture insert with 0.4-μm-sized pores (Merck) placed on 6-well plates (Greiner). After reaching confluence, the monolayers were stimulated for 48 h with 3 mM sodium butyrate (Sigma-Aldrich) in serum-free medium. Conditioning of differentiated HT-29 monolayers by *Blastocystis* was performed for 24 h at 37 °C in anaerobic condition. Incubation of the HT-29 monolayers with *B. longum* was performed for 6 h for viability determination. Transepithelial electrical resistance across the monolayers was measured using Millipore-ERS-2 volt-ohm-meter. Flux assay was performed using fluorescein isothiocyanate-conjugated Dextran 4000 (FITC-Dextran) (Sigma). The assay included washing of the monolayer on the inserts twice with Hank’s balanced salt solution (HBSS) (Thermofisher). FITC-Dextran at a concentration of 100 μg/ml in HBSS was added on the apical compartment, and the plate was incubated at 37 °C. The buffer at the basolateral compartment was collected after 1 h and transferred to a black 96-well plate (Nunc). Fluorescence was measured using Tecan Infinite F200 microplate reader at excitation and emission wavelengths of 492 nm and 518 nm, respectively.

### Acute infection of *Blastocystis* in a mouse model

The animal experiments were performed according to the Singapore National Advisory Committee for Laboratory Animal Research guidelines. The protocol (R13-5890) was approved by the NUS Institutional Animal Care and Use Committee. The infection of *Blastocystis* into mice was carried out according to a previous protocol [30]. C57BL/6 male mice, aged 5 to 6 weeks, were given 2% DSS in drinking water for 4 days followed by a recovery period of 5 days. After the recovery period, they were injected with 5 × 10^7^ live *Blastocystis* cells intracecally. The mice were subjected to anesthesia (ketamine 75 mg/kg + medetomidine 1 mg/kg via intraperitoneal (IP) injection) then a vertical incision was made on the abdomen. The cecum was exteriorized, and 50 μl *Blastocystis* suspended in PBS was injected into the caecum using a 27G needle. Sham surgical controls were injected with 50 μl PBS intracecally. The incision was then closed with two layers of sutures. Subsequently, anesthesia was reversed (Atipamezole 1 mg/kg via subcutaneous (SC) injection), and antibiotics (Enrofloxacin10 mg/kg SC) and analgesic (Carprofen 5 mg/kg SC) were given. Fecal samples were collected at various time-points—before surgery at day 0, day 1 post-infection, day 2 post-infection, and day 3 post-infection. A total of 24 mice were included, with 8 mice in each of the 3 groups—control, ST7-B-infected and ST7-H-infected. The rest of the mice were euthanized on day 3 post-infection. The colon and cecum were extracted for histology.

### Determination of bacterial abundance in mice fecal samples

DNA from 42 mg each of mouse fecal samples were extracted using QIAamp Fast DNA Stool Mini Kit (Qiagen) following the manufacturer’s instructions. The relative abundance of selected bacterial groups was determined in a qPCR assay using the DNA primers listed (Table 1). SsoAdvanced™ Universal SYBR Green Supermix (Bio-Rad) was used, and amplifications were carried out in an iCycler thermocycler with iQ5 attachment (Bio-Rad).

**Table 1:** DNA primers used for qPCR assay to determine the relative abundance of bacterial group in mice fecal samples

Target	Sequences (5′ → 3′)	Reference
*16S rRNA* (total bacteria)		[35]
Forward	ACTCCTACGGGAGGCAGCAGT	
Reverse	GTATTACCGCGGCTGCTGGCAC	
*Bacteroides* sp.		[35]
Forward	GTCAGTTGTGAAAGTTTGC	
Reverse	CAATCGGAGTTCTTCGTG	
*Lactobacillus* sp.		[36]
Forward	AGCAGTAGGGAATCTTCCA	
Reverse	CACCGCTACACATGGAG	
*Bifidobacterium* sp.		[35]
Forward	AGGGTTCGATTCTGGCTCAG	
Reverse	CATCCGGCATTACCACCC	
*E. coli*		[35]
Forward	CATGCCGCGTGTATGAAGAA	
Reverse	CGGGTAACGTCAATGAGCAAA	

### Statistical analysis

Comparisons of two groups were done using Student’s *t* test for paired samples. Comparisons of more than two groups were done using analysis of variance (ANOVA). Analyses and generation of graphs were done using Prism GraphPad version 5.

## Results

### Gut bacteria exerted positive effects on *Blastocystis* cell count in vitro

**Fig. 1:**
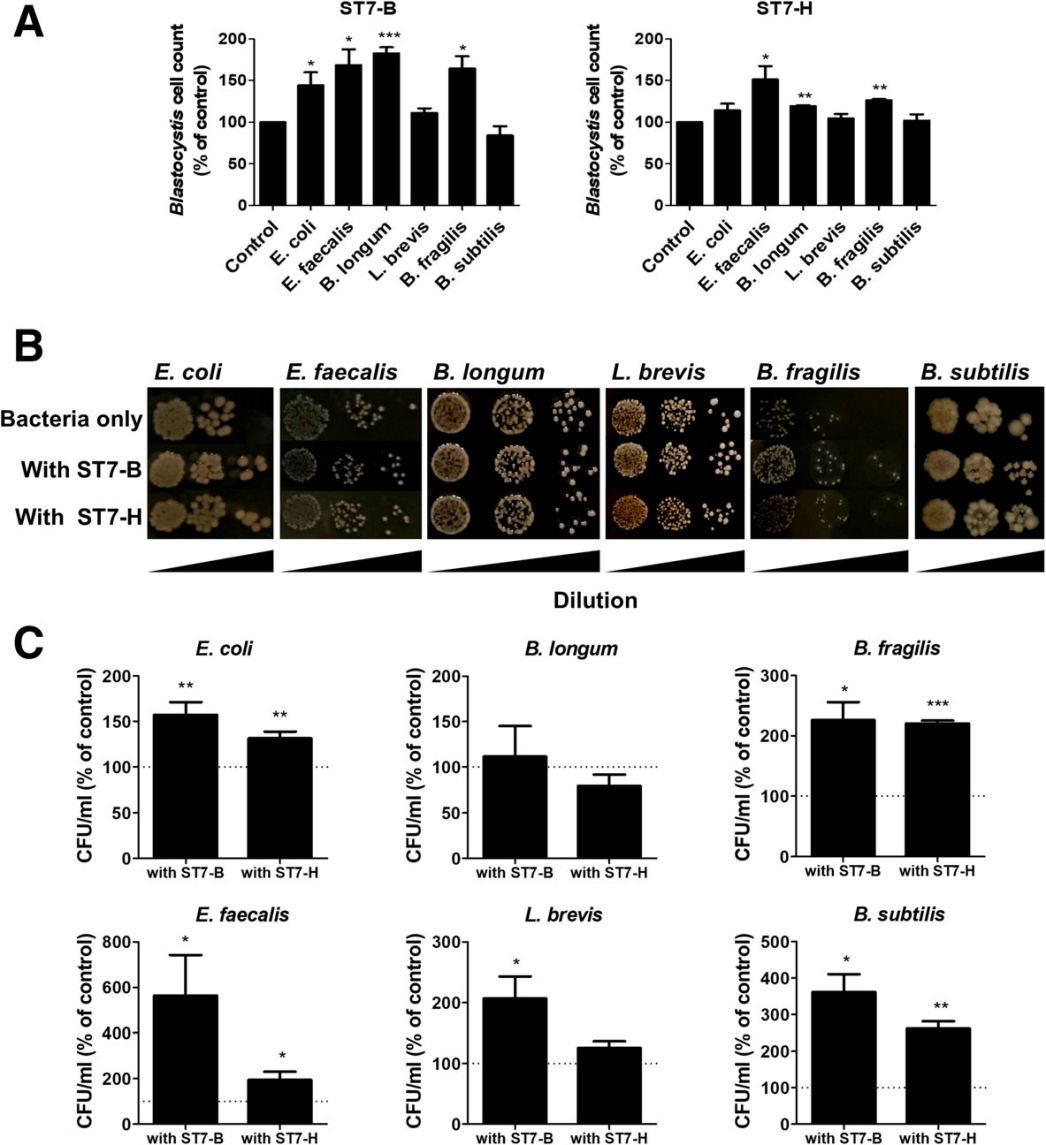
Interactions of *Blastocystis* with representatives of gut bacteria. *Blastocystis* ST7 isolates B and H were incubated for 24 h at 37 °C in PBS with each of the following bacterial cultures: *E. coli*, *E. faecalis*, *B. longum*, *L. brevis*, *B. fragilis*, and *B. subtilis*. There were higher counts of *Blastocystis* when incubated with bacteria (**a**). The highest increase in *Blastocystis* count was observed between isolate ST7-B and *B. longum*. The representative gut bacteria also had higher colony-forming unit per milliliter as observed (**b**) and counted (**c**) from agar plates, except for *B. longum*. *B. longum*’s colony-forming unit per milliliter was not significantly different in the presence of *Blastocystis* (**c**). *p < 0.05; **p < 0.01; ***p < 0.001

To determine whether the presence of gut commensal bacteria affects *Blastocystis* cell count in vitro, ST7-H and ST7-B were individually co-incubated with representative bacteria of the gut microbiota—*E. coli*, *E. faecalis*, *B. longum*, *L. brevis*, *B. fragilis*, and *B. subtilis*. The reduced PBS condition used for co-incubation ensured a low oxygen environment necessary for *Blastocystis* viability while the simple PBS formulation minimizes potential exogenous growth factors that would otherwise complicate the assay, resulting in bacterial overgrowth. Generally, both ST7-B and ST7-H displayed higher cell counts when co-incubated with gut commensal bacteria, with differential effects observed depending on the species of bacteria (Fig. 1a). More specifically, significant positive effects were observed when ST7-B was co-incubated with *E. coli*, *E. faecalis*, *B. longum*, and *B. fragilis*, and when ST7-H was co-incubated with *E. faecalis*, *B. longum*, and *B. fragilis*. The highest observed positive effect was observed between ST7-B and *B. longum.*

### *Blastocystis* exerted positive effects on some gut bacteria in vitro

The CFU counts of *E. coli*, *E. faecalis*, *L. brevis*, *B. longum*, *B. subtilis*, and *B. fragilis* were also examined when they were co-incubated with *Blastocystis* ST7-B and ST7-H. Gut commensal bacteria colony-forming unit per milliliter values were generally higher when co-incubated with *Blastocystis* cells. Representative images of the bacterial colonies on agar plates for the co-incubation assay are shown (Fig. 1b). *E. coli*, *E. faecalis*, *B. fragilis*, and *B. subtilis* had significantly higher CFU count when co-incubated with both ST7-B and ST7-H (Fig. 1c). *L. brevis* also had higher CFU count after co-incubation, but this was only significant for ST7-B. Interestingly, *B. longum* displayed lower CFU count when co-incubated with ST7-H. Average CFU count was moderately higher when co-incubated with ST7-B, but the differences did not reach statistical significance. An overall greater growth effect on CFU count was observed when ST7-B was co-incubated with gut commensal bacteria compared to ST7-H.

### *Blastocystis* positively affects *E. coli* and negatively affects *B. longum* in a three-way co-culture setup

**Fig. 2:**
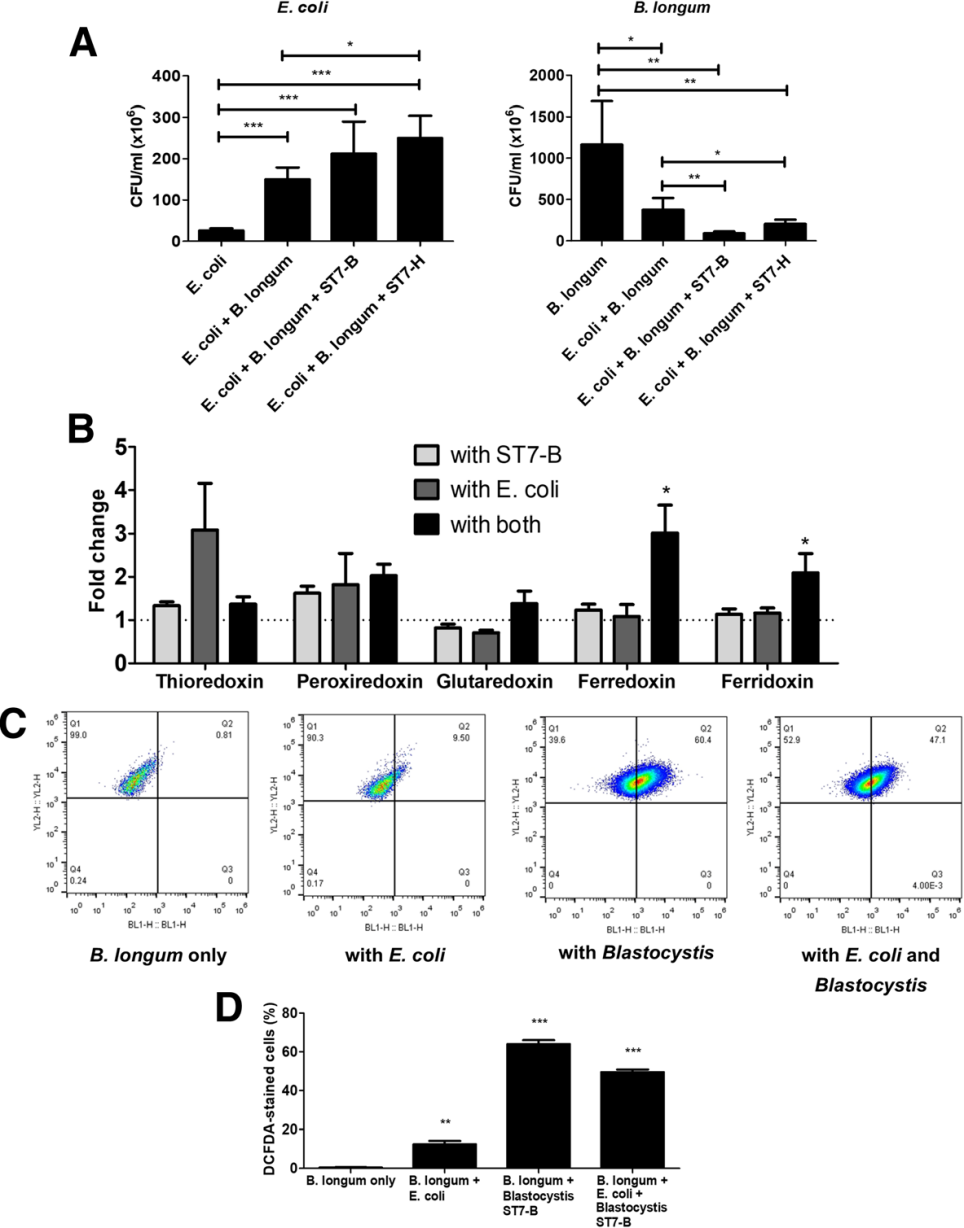
*Blastocystis* inhibition of *B. longum* is linked to an increase in cellular ROS. *B. longum* exhibited lower colony-forming unit per milliliter when incubated with *E. coli* for 24 h at 37 °C in PBS. The count is even lower in the presence of *Blastocystis* (**a**). There is an increase in some oxidoreductase genes in *B. longum* when it is incubated with *E. coli* and *Blastocystis* indicating that the bacterium is under oxidative stress (**b**). Flow cytometry analysis shows *B. longum* cells’ shift to the right indicating more cells have cellular ROS content when co-incubated with *E. coli*, *Blastocystis*, or both (**c**). *Blastocystis* caused a greater increase in ROS content compared to *E. coli* (**d**). *p < 0.05; **p < 0.01; ***p < 0.001

To investigate further if the effect of *Blastocystis* on gut bacteria is selective, a co-culture arrangement involving *Blastocystis*, *E. coli*, and *B. longum* was prepared. After incubation, *E. coli* had significantly higher colony-forming unit per milliliter compared to controls when incubated with *B. longum*. The presence of *Blastocystis* further increased the CFU of *E. coli* (Fig. 2a). On the other hand, *B. longum* displayed significantly lower colony-forming unit per milliliter compared to controls when incubated with *E. coli*, which was further reduced by both *Blastocystis* ST7-B and ST7-H (Fig. 2a).

### *E. coli* and *Blastocystis* caused oxidative stress to *B. longum*

As oxidative stress is a known contributor to dysbiosis [37], we explored whether *Blastocystis* and *E. coli* was impacting the viability of *B. longum* via such a mechanism. After incubation with *Blastocystis* and *E. coli*, the oxidoreductase gene expression of *B. longum* was analyzed. Results showed that two of the oxidoreductase genes, ferredoxin and ferridoxin, were upregulated when *Blastocystis* and *E. coli* were present (Fig. 2b). This suggests that the bacterium is undergoing oxidative stress in the presence of the organisms mentioned. Flow cytometry analysis of ROS content demonstrated that *Blastocystis*, *E. coli*, or both caused more *B. longum* cells to convert DCFDA stain indicating the presence of cellular ROS (Fig. 2c). Interestingly, the presence of *Blastocystis* alone caused significantly more production of ROS in *B. longum* than in combination with *E. coli* (Fig. 2d).

### *B. longum* protects intestinal epithelial barrier against *Blastocystis*-induced damage

**Fig. 3:**
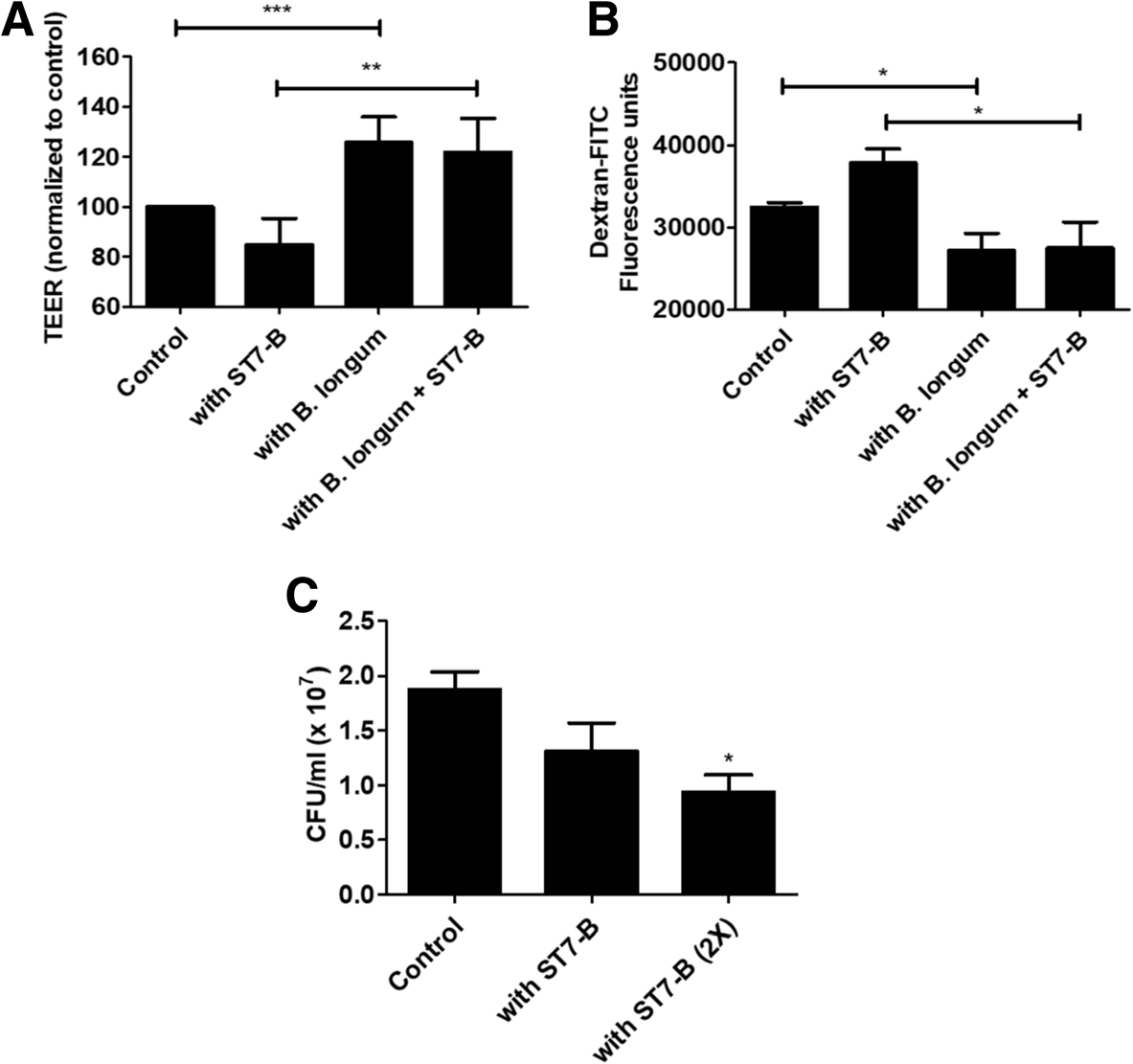
Host responses to *Blastocystis*-*B. longum* interaction. *B. longum* had a protective effect on the integrity of the epithelial barrier as observed on HT-29 monolayers incubated with *Blastocystis*. Monolayers had higher transepithelial electrical resistance values when *B. longum* is present (**a**) even in the presence of *Blastocystis*. Likewise, flux assay showed less FITC-Dextran molecules pass through the layer when *B. longum* is present (**b**). However, *Blastocystis*-induced host responses have a negative effect on *B. longum* as shown by the lower bacterial counts when this bacterium was incubated with *Blastocystis*-primed HT-29 monolayer (**c**). *p < 0.05; **p < 0.01; ***p < 0.001

To determine the significance of *B. longum* for the host, HT-29 monolayers were grown and incubated with *Blastocystis* and *B. longum*. TEER measurements showed that *B. longum* can help maintain the epithelial barrier as observed from higher TEER compared to controls (Fig. 3a). Flux assays using FITC-dextran also showed that less number of reporter molecules can pass through the barrier when *B. longum* is present (Fig. 3b). Both assays showed that *B. longum* is beneficial to the host in maintaining the barrier and even negating the damage caused by *Blastocystis*. However, host factors induced by *Blastocystis* could inhibit *B. longum* growth. This is shown in co-culture assays whereby HT-29 monolayers were previously conditioned by *Blastocystis*. In these test wells, the colony-forming unit per milliliter count of *B. longum* was significantly lower than the control (Fig. 3c).

### *Blastocystis*-infected mice had lower *Bifidobacterium* sp. and *Lactobacillus* sp. but higher *E. coli* abundance in the fecal samples

**Fig. 4:**
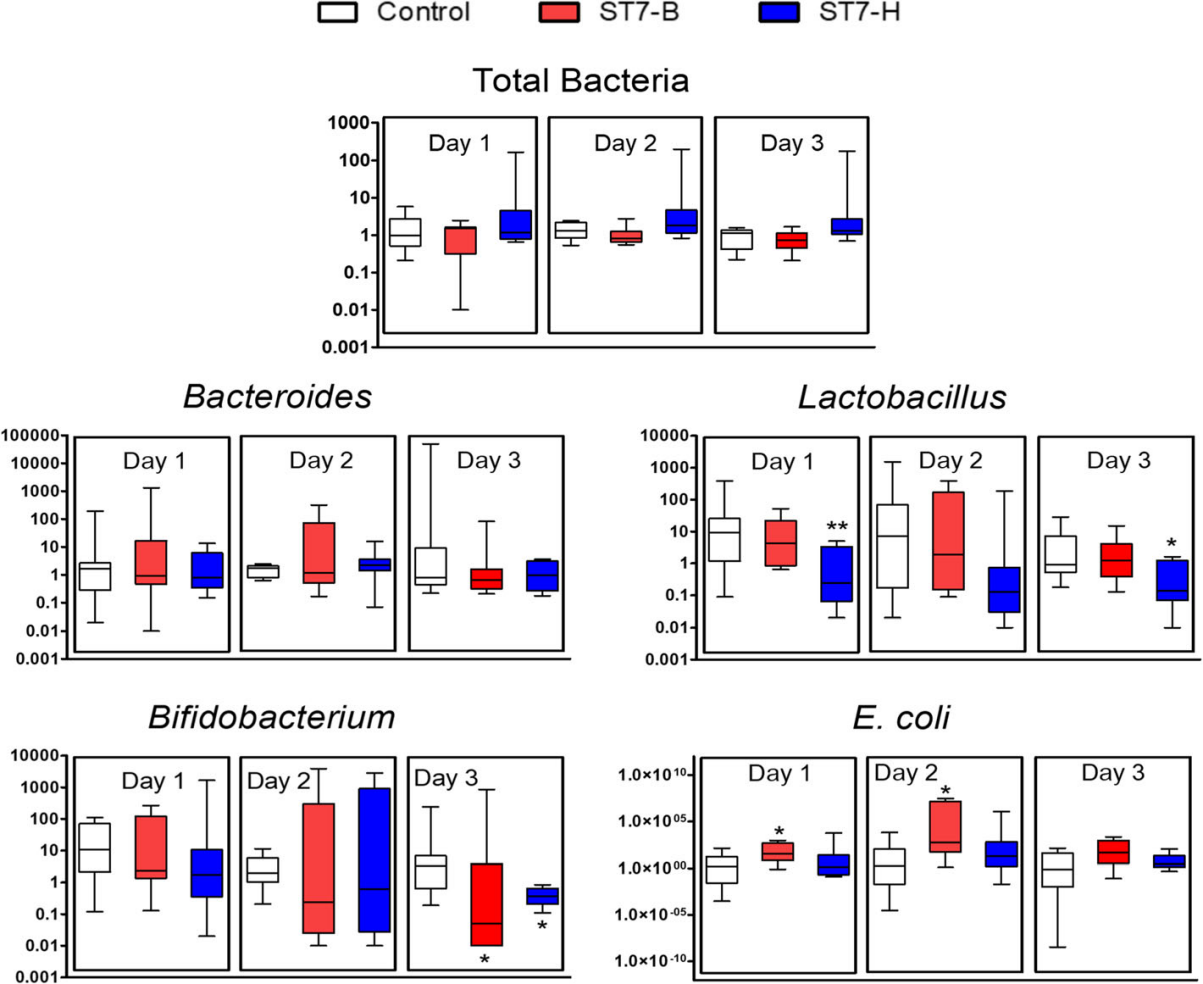
Relative abundance of representatives of gut bacteria in mouse fecal samples. The mice were surgically infected with *Blastocystis* ST7-B and ST7-H isolates, and fecal samples were collected before surgery and 1-, 2-, and 3-day post infection. The DNA was extracted from 42 mg of samples and gut bacteria 16S rDNA genes were detected in qPCR. Analyses showed an increase in *E. coli* in mice infected with *Blastocystis* ST7-B at 1- and 2-day post infection. On the other hand, there was a decrease in *Lactobacillus* and *Bifidobacterium* in *Blastocystis*-infected mice. *Blastocystis* ST7-H isolated caused a decrease in *Lactobacillus* in mice after 1 and 3 days of infection. *Blastocystis* ST7-B and ST7-H caused a decrease in *Bifidobacterium* after 3 days of infection. *p < 0.05; **p < 0.01

To determine whether *Blastocystis* infection alters the gut microbiota after acute infection in mice, fecal pellets were collected from various time-points before and after *Blastocystis* infection. The fecal pellets were subjected to qPCR to quantify the relative abundance of total bacteria, *Bacteroides*, *Lactobacillus*, *Bifidobacterium*, and *E. coli*. Bacterial relative abundance in the mouse fecal samples at equal weight was compared between the *Blastocystis*-infected mice with the control mice. The values were first normalized to the relative abundance found at their respective conditions before surgery. There was little to no difference in total bacteria between controls and *Blastocystis*-infected mice (Fig. 4). However, significant reduction in *Bifidobacterium* sp. was observed on day 3 after infection with *Blastocystis* ST7-B or ST7-H. Significant reduction in *Lactobacillus* sp. was also observed but only in fecal samples of ST7-H-infected mice at days 1 and 3 post-infection. *E. coli* had significantly higher abundance in ST7-B-infected mice on both days 1 and 2 post-infection. These observations suggest that, in general, *Blastocystis* can selectively influence gut microbiota populations, and in this case, could negatively affect beneficial bacterial populations.

### Histopathology examination revealed tissue damage in the colon of *Blastocystis*-infected mice

**Fig. 5:**
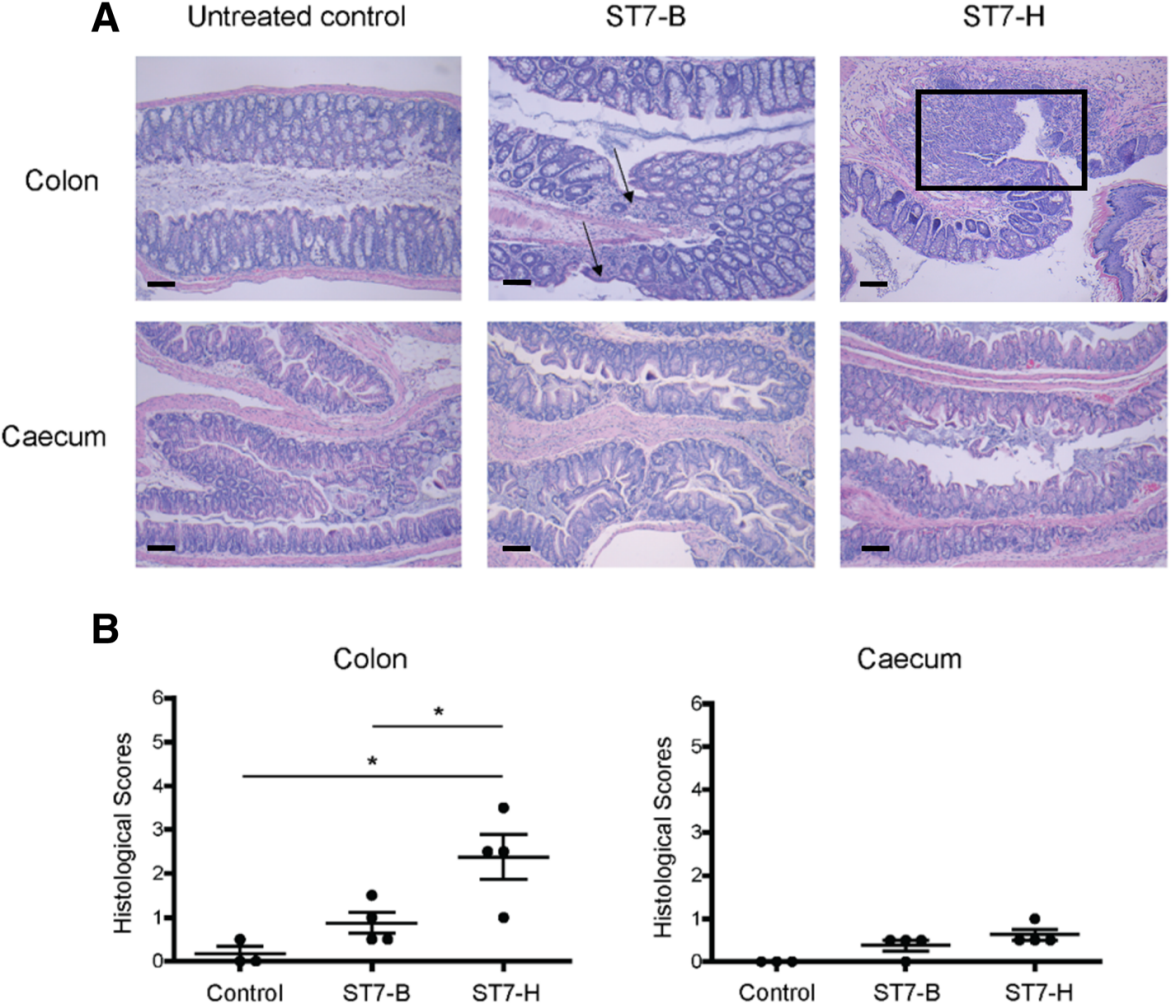
Histological examination of mouse tissue after *Blastocystis* infection. **a** Histology images show the tissues with the highest histological scores within each treatment group. Epithelial lesions/ulcers and loss of crypt architecture were observed for the colon of mice infected with ST7-B (black arrows) and ST7-H (boxed area). Scale bar = 100 μm. **b** Dot plots show the histological scores for individual mice. Histological scores for ST7-H were significantly higher than that of ST7-B and the control in the colon. *p < 0.05

To further determine the effect of *Blastocystis* in mouse intestinal tissue, a histopathological examination and scoring was done on mouse colon and cecum tissue. The results showed damage and ulceration in the colon from *Blastocystis*-infected mice (Fig. 5a), with significantly higher pathological scores in the colon tissue of ST7-H-infected mice compared to control mice (Fig. 5b). The cecum did not appear to be damaged by *Blastocystis*.

## Discussion

Although previous studies have reported associations between *Blastocystis* and gastrointestinal disorders, the protist’s pathogenic potential and clinical significance still remains to be established [38]. To address the issue of *Blastocystis*’ pathogenesis, it could be useful to determine whether *Blastocystis* colonization is associated with gut dysbiosis, which is known to affect intestinal health [4]. Various epidemiological studies have been executed in the past to investigate the associations between *Blastocystis* and dysbiosis, with conflicting results obtained [21–25, 39, 40]. One important limiting factor of some of these previous studies was that the subtype of *Blastocystis*, which has variations in terms of pathogenic potential, was not controlled for or identified [41, 42]. This study was therefore conducted with the aim of using a specific subtype (ST7) to study *Blastocystis*-gut bacteria interactions and to determine whether *Blastocystis* infection could disrupt the gut microbiota in vitro and in vivo.

Two *Blastocystis* ST7 isolates, ST7-B and ST7-H, were used in this study since previous reports indicate their pathogenic potential. In vitro assays on ST7 revealed that the isolate caused disruptions in the gut epithelial barrier by disrupting tight junction proteins such as occludin and zonula occludens-1 (ZO-1), and also have greater adhesiveness than ST4 isolates to intestinal epithelial cells [29, 43]. Furthermore, ST7 was shown to have significantly greater cysteine protease activity compared to ST4 [29]. *Blastocystis* ST7 have been shown to be more resistant to anti-parasitic drugs [28, 44] and against the host innate immune response [45] compared to ST1 and ST4 isolates. *E. coli*, *E. faecalis*, *B. longum*, *L. brevis*, *B. fragilis*, and *B. subtilis* were chosen for the co-incubation assay as representative species of the gut microbiota [46–49]. Among these, *L. brevis* and *B. longum* are well-known probiotic species which contribute protective benefits for the gut [49, 50]. More importantly, they have been found to improve intestinal conditions through the mitigation of IBD and IBS [51]. Although the other species (*E. coli*, *E. faecalis*, *B. fragilis*, *B. subtilis*) are not widely considered as probiotic, they are still important commensal gut bacteria, playing roles in carbohydrate metabolism, production of important metabolites, and the exclusion of potential pathogens [52]. Changes in the viability of these bacterial species (represented by CFU count in the assay) in the presence of *Blastocystis* may lead to potential disruptions in the microbiota. This may have important implications in relation to IBD and IBS.

The in vitro co-incubation assay demonstrated that *Blastocystis* cell count was higher in the presence of gut bacteria (Fig. 1a). It is possible that the bacteria secretory products or dead cells in the suspension act as a nutrient source for *Blastocystis*, allowing it to survive better when incubated in PBS. It is still unclear how *Blastocystis* obtains nutrients, but the mechanisms involved can be speculated. Although *Blastocystis* does not have true mitochondria, it has been found to possess mitochondrion-derived double-membrane-bound organelles called mitochondrion-like organelles (MLOs) [53]. These MLOs are likely hydrogenosomes, which are found in “amitochondriate protists,” which can play roles in carbohydrate and amino acid metabolism. Within the MLOs, two key enzymes involved in anaerobic energy metabolism have been identified, namely, pyruvate:ferredoxin oxidoreductase (PFO) and [FeFe] hydrogenase [54]. PFOs function in carbohydrate metabolism, catalyzing the conversion of pyruvate to acetyl-CoA and CO_2_ [55]. [FeFe] hydrogenases function in hydrogen metabolism [56]. These two enzymes may be activated in the presence of bacterial products, which may be utilized as nutrient sources for *Blastocystis*. In comparison, *Blastocystis* cells in the control may have been starved of nutrients when they are incubated in PBS. The co-incubation assay also demonstrated higher bacterial CFU count when gut commensal bacteria were co-incubated with *Blastocystis* (Fig. 1b, c). Higher bacterial CFU count may be a result of bacteria breaking down dead cells (from both *Blastocystis* and existing bacteria cells), in order to obtain a nutrient source in the PBS suspension. It was also observed that there were differential effects across bacterial species, with *E. coli*, *E. faecalis*, *B. subtilis*, and *B. fragilis* displaying more prominent positive effects compared to *L. brevis* and *B. longum* after co-incubation with *Blastocystis*. *Blastocystis* and gut commensal bacteria generally exhibit a mutualistic relationship when co-incubated in vitro, evidenced by higher parasite numbers and bacterial CFU counts after co-incubation. *E. coli*, *E. faecalis*, *B. fragilis*, and *B. subtilis* appeared to have more significant positive effects than *L. brevis* and *B. longum*. This observation suggests that these bacteria received less beneficial effects and may have a weaker mutualistic relationship with ST7-B and ST7-H in vitro. Overall, the co-incubation assays show that *Blastocystis* can interact with gut-commensal bacteria, which may lead to changes in the gut microbiota. Furthermore, there may be differential interactions depending on the species of bacteria present. Hence, this served as a basis for the subsequent in vivo assays.

A three-way setup involving *Blastocystis*, *E. coli*, and *B. longum* was used to further investigate if *Blastocystis* had a selective influence on specific groups of gut bacteria. Results showed that *Blastocystis* could boost the growth of *E. coli* while inhibiting *B. longum* (Fig. 2a). *E. coli* is a facultative anaerobe while *B. longum* is an obligately anaerobe. These interactions suggest oxidative stress as a factor in the experimental outcome [57]. Indeed, gene expression analysis of *B. longum* showed that some of the bacterium’s oxidoreductase genes are upregulated, suggesting that it is undergoing oxidative stress in the presence of *Blastocystis* and *E. coli* (Fig. 2b). In addition, a greater percentage of *B. longum* cells exhibited cellular ROS content when these were incubated with *Blastocystis* and *E. coli* (Fig. 2c, d). Our results, however, do not exclude other mechanisms of *Blastocystis*- and *E. coli-*mediated killing of *B. longum*. Possible implications of redox-mediated killing of obligate anaerobes would be decreased diversity in gut bacteria ultimately leading to dysbiosis [58].

**Fig. 6:**
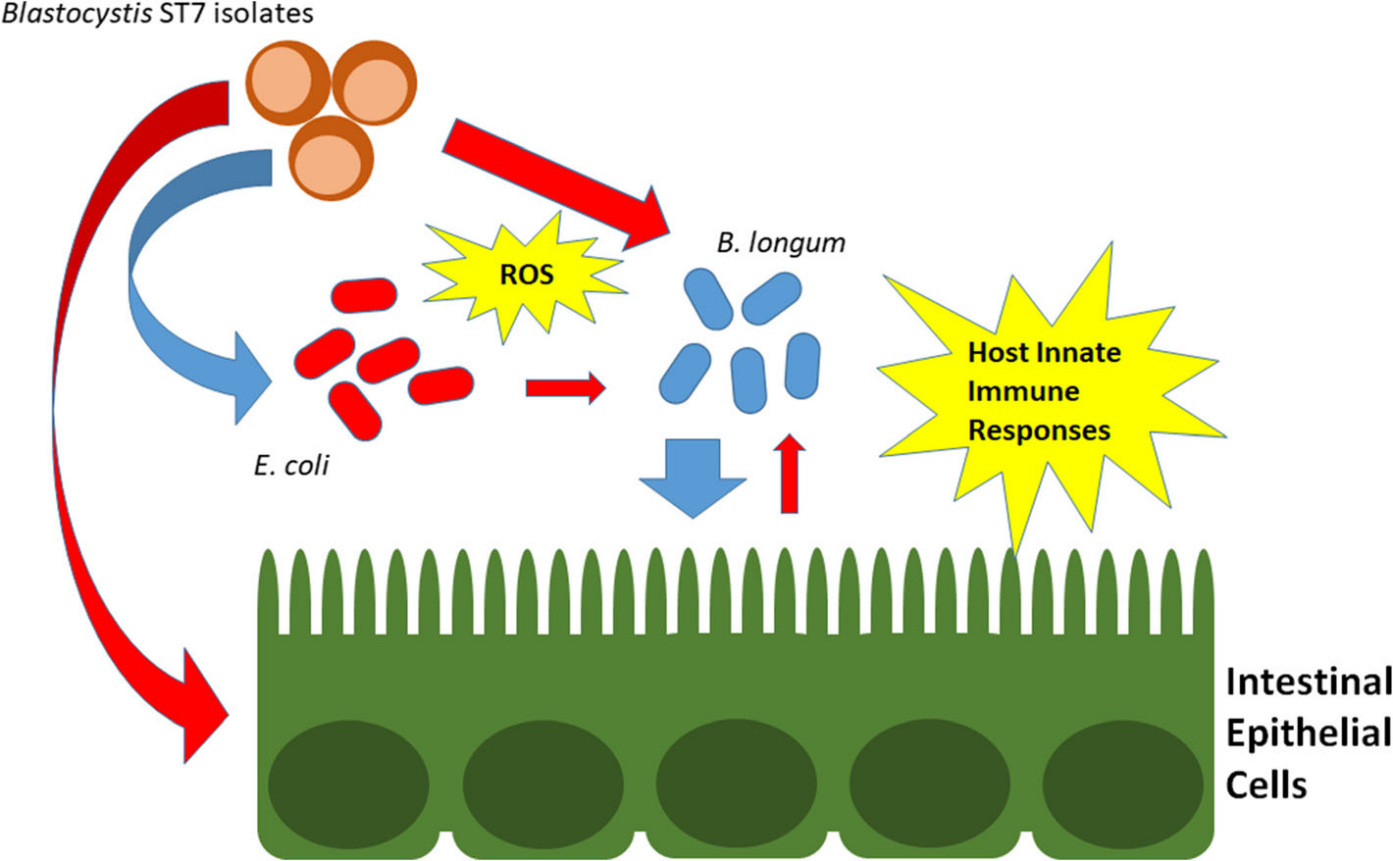
Interactions of *Blastocystis* with gut bacteria and the effect on the host. *Blastocystis* could disrupt gut microbiota selectively. In this study, *Blastocystis* caused reduction of *B. longum* but an increase in *E. coli*. This could happen by several mechanisms. There is a direct effect of *Blastocystis* through oxidative stress, limiting the viability of obligately anaerobic bacteria. Host immune responses as induced by *Blastocystis* could also limit *Bifidobacterium*. This bacterium is important to protect the epithelial barrier from *Blastocystis*-mediated damage. Red and blue arrows signify negative and positive interactions respectively

The significance of *B. longum* in the context *Blastocystis* infection was explored using intestinal epithelial monolayer assays. Specifically, the role of *B. longum* as well as the effect of *Blastocystis* on the epithelial barrier integrity was investigated using TEER measurements and flux assay. These in vitro assays showed that *B. longum* helps to maintain the intestinal epithelial barrier (Fig. 3a, b), even in the presence of *Blastocystis*. This supports the notion that *B. longum* and similar groups of gut bacteria are essential for the health of the gut [51]. Past studies reported that the presence of *Bifidobacterium* attenuated the decrease in transepithelial electrical resistance and increase in paracellular permeability in Caco-2 cells treated with LPS. *Bifidobacterium* was also found to upregulate the expression of tight junction proteins occludin, claudin-3, and ZO-1 as well as aid the localization of these proteins to the epithelial tight junctions [58]. Aside from maintaining the epithelial barrier, *Bifidobacterium* can also exert anti-inflammatory properties as it can reduce the production of pro-inflammatory cytokines IL-6 and TNF-α [58]. In contrast, *Blastocystis* ST7 disrupts tight junction proteins such as occludin and ZO-1 [43, 59] as well as increases the levels of pro-inflammatory cytokines to trigger an inflammatory response [60, 61]. These show that *Bifidobacterium* can potentially negate the cytopathic effects of *Blastocystis* on the hosts. However, host-secreted factors that result from *Blastocystis* infection could be limiting to *Bifidobacteria*, as shown from the co-culture assays involving HT-29 cells previously conditioned by *Blastocystis* (Fig. 3c). *Blastocystis* therefore may not only affect *Bifidobacteria* directly but could also limit the bacterium through the host. These host factors may include elements of the innate immunity such as antimicrobial peptides. We have previously shown that *Blastocystis* can induce intestinal epithelial cells to secrete LL-37, a fragment of cathelicidin with antimicrobial properties [45]. These factors, however, have broad effects that do not only affect invading pathogens but could also impact local microbial populations when overly secreted. This added pressure could therefore result in lower diversity of microbial populations in the gut. Overall, the *Blastocystis*-*Bifidobacterium*-host epithelial cell interactions are complex and could involve numerous signaling and effector molecules. Our study reveals that ROS and host factors may play roles in limiting *B. longum* viability, providing new clues on how *Blastocystis* influences specific gut microbiota populations (Fig. 6).

In this study, an acute infection of *Blastocystis* ST7-B and ST7-H on mice was performed to assess *Blastocystis-*induced changes in the gut microbiota using qPCR (Fig. 4). This study utilized a DSS colitis mouse model, which improves *Blastocystis* colonization rates, as previously demonstrated for ST7-B- and ST7-H-infected C57BL/6 mice treated with low concentrations of DSS [27]. Total bacteria levels and the relative abundance of *Bacteroides*, *Lactobacillus*, *Bifidobacterium*, and *E. coli* populations were quantified using qPCR after *Blastocystis* infection. The reduction was observed in *Bifidobacterium* in mice infected by ST7-B and ST7-H. There was also lower abundance observed in *Lactobacillus* in ST7-H-infected mice. Interestingly, there was a higher abundance of *E. coli* in ST7-B-infected mice. These results are in concordance with what has been obtained using in vitro assays; *Bifidobacterium* was reduced, and *E. coli*’s abundance increased. In the case of *Lactobacillus*, its reduction was only observed in vivo. It is possible that host factors, which are not present in the in vitro assays, come into play in the observed reduction of *Lactobacillus*. Histological examination of mouse tissues (Fig. 5) corroborated with our previous study [30]. The pathology scoring also identified ST7-H as more able to cause tissue damage than ST7-B. However, in this study, ST7-B appeared to be a better driver of dysbiosis than ST7-H. These observations point to differences between the isolates’ mechanism of pathogenesis, with ST7-H causing more direct damage to host cells and ST7-B causing harm through dysbiosis.

Together with *Bifidobacterium* spp. discussed above, *Lactobacillus* is another group of probiotic bacteria in the gut. Like *Bifidobacterium*, members of this genus also have similar anti-inflammatory properties [62]. An in vitro study showed that *L. casei* could reduce T cell response by dendritic cells in healthy and ulcerative colitis patients, thus decreasing the inflammation. This is achieved through increased production of IL-4 and decreased secretion in IL-22 and IFN-γ [63, 64]. *Lactobacillus* has also been found to significantly increase IgA levels [65]. Several in vivo studies using the DSS colitis mouse model showed that administration of both *Lactobacillus* and *Bifidobacterium* improved clinical symptoms of colitis and enhanced mucus production [66, 67]. Hence, a reduction in both gut bacteria would remove an element of protection from the gut epithelium, aiding the pathogenesis of *Blastocystis*. This could explain the intestinal tissue damage seen in the histology results of the current study. Epidemiological studies have also shown that reductions in these two bacteria could increase the susceptibility to gastrointestinal disorders. UC and CD patients were found to possess lower levels of *Lactobacillus* and *Bifidobacterium* populations, respectively [68, 69]. Similarly, *Lactobacillus* and *Bifidobacterium* levels are lower in IBS patients than that in healthy controls [70, 71]. Hence, the presence of *Blastocystis* ST7 could cause disease not only just directly but also through reduction of beneficial bacteria.

Various epidemiological studies have been conducted to investigate the links between *Blastocystis* and dysbiosis. Previous surveys have observed certain characteristics in the microbiota of *Blastocystis*-positive subjects, leading to the association of *Blastocystis* with a healthy human gut [21–24, 27]. A study found that *Blastocystis*-positive individuals free from IBD had higher fecal bacterial diversity, higher abundance of Clostridia, and lower abundance of Enterobacteriaceae [22]. A recent study done across 12 metagenomic datasets found a strong association between *Blastocystis* and the enrichment of Firmicutes and Clostridiales, as well as the reduction in Bacteroides [21]. Additionally, another group showed that *Blastocystis* is linked to a healthy gut, based on the high *F. prausnitzii–E. coli* ratio in *Blastocystis*-positive subjects [23]. These and other studies formed the basis for asserting that *Blastocystis* is a member of the normal, healthy gut microbiota [72, 73]. It is important to note that two of the mentioned studies did not identify the subtype of *Blastocystis* present in its subjects [22, 23]. For the study which did identify the subtypes, a whole array was found in the subjects, including ST1 and ST3 which are associated with asymptomatic infections [21]. The subjects from these studies could be predominantly colonized with subtypes associated with lower pathogenic potential and may be associated with healthy gut microbiota. However, this present study used a specific subtype, ST7, which exhibits high pathogenic potential. Therefore, the conflicting results may be due to the differential effects of various subtypes on the host and gut microbiota. It is therefore important for future studies to control or stratify for *Blastocystis* subtypes to avoid self-limiting results. The results of this study are in line with one epidemiological study, which concluded that *Blastocystis* is linked to dysbiosis [25]. In that study, *Blastocystis*-positive patients with IBS-C had a significant decrease in *Bifidobacterium* and *Lactobacillus* populations. It was also noted that the subtype of *Blastocystis* involved in the study by Nourisson et al. was predominantly ST4. Compared to other STs except ST7, ST4 has a moderate pathogenic potential [29], and this could be the reason that *Blastocystis* was associated with dysbiosis, unlike the other studies. Overall, the findings from Nourisson et al. corroborate with the results of this study, both suggesting that virulent subtypes of *Blastocystis* are more likely to be associated with dysbiosis, and its pathological outcomes, including IBD and IBS.

## Conclusion

Overall, this study investigated the interactions of pathogenic isolates of *Blastocystis* ST7 with known members of the gut microbiota. To our knowledge, this is the first time wherein in vitro setups complemented by an in vivo system were utilized to investigate the interactions of *Blastocystis* with the gut microbiota. In addition, this study also focused on a specific ST of *Blastocystis*. While most reports on *Blastocystis* label it as a commensal and a member of healthy gut microbiota, the findings in this study indicate that different ST of *Blastocystis*, represented by two pathogenic isolates, may modulate gut microbiota differently from more common STs (e.g., ST1–3). Future work should include other *Blastocystis* STs with lesser pathogenic potential as well as involving more representatives of gut bacteria. This should provide a clearer picture on where *Blastocystis* and its STs really stand on gut health and disease.
